# Down Syndrome: how to communicate the diagnosis

**DOI:** 10.1186/s13052-023-01419-6

**Published:** 2023-02-09

**Authors:** Caterina Gori, Guido Cocchi, Luigi Tommaso Corvaglia, Giuseppe Ramacieri, Francesca Pulina, Giacomo Sperti, Valeria Cagnazzo, Francesca Catapano, Pierluigi Strippoli, Duccio Maria Cordelli, Chiara Locatelli

**Affiliations:** 1 IRCCS Institute of Neurological Sciences of Bologna, UOC Neuropsychiatry of the Pediatric Age, Bologna, Italy; 2 Department of Medical and Surgical Sciences (DIMEC), Neonatology Unit, St. Orsola‑Malpighi Polyclinic, University of Bologna, Via Massarenti 9, 40138 Bologna, BO Italy; 3 Department of Medical and Surgical Sciences (DIMEC), University of Bologna, Via Massarenti 9, 40138 Bologna, BO Italy; 4 Department of Developmental Psychology and Socialisation, University of Padova, Via Venezia 8, 35131 Padua, PD Italy; 5 Speciality School of Paediatrics – Alma Mater Studiorum, University of Bologna, Via Massarenti 9, 40138 Bologna, BO Italy; 6 Unit of Histology, Embryology and Applied Biology, Department of Experimental, Diagnostic and Specialty Medicine (DIMES), University of Bologna, Via Belmeloro 8, 40126 Bologna, BO Italy; 7 IRCCS, University Hospital of Bologna St. Orsola-Malpighi Polyclinic, Neonatology Unit, Via Massarenti 9, 40138 Bologna, BO Italy

**Keywords:** Down Syndrome, Communication, Prenatal diagnosis, Postnatal diagnosis, Decision-making process

## Abstract

Communicating the diagnosis of Down Syndrome to a couple of parents is never easy, whether before or after birth. As doctors, we must certainly rely on our own relational skills, but it is also necessary to be confident in some general indications, which are often overlooked in the strict hospital routine. This article is intended as a summary of the main articles published on this subject in the international literature, collecting and summarising the most important indications that have emerged in years of medical practice all over the world as well as in our personal experience. The diffusion of these guidelines is essential to help the doctor in this difficult task, on which there is often little training, and above all to guarantee to the parents the least traumatic communication possible.

## Introduction

The information about a suspected Down syndrome’s (DS) diagnosis, given either before or after birth, has a profound effect on parents [6]. At present, most families report a degree of dissatisfaction with the way in which doctors told them the diagnosis, with the information they were given, which was often described as outdated and overly negative, and with the support they received afterwards [14, 24]. As professionals it is therefore our responsibility to inform and prepare ourselves for the communication of these diagnoses, and to date there are specific recommendations to guide doctors on this sensitive issue. The purpose of this work is to summarize the most important indications on this subject in order to provide doctors with useful guide and thus, ensure proper communication to patients enabling them to establish a good parent-child and doctor-patient relationship.

### When to communicate the diagnosis?

The physician should prepare to communicate the diagnosis as soon as it is reached and, according to several studies, it is preferable to inform parents when the diagnosis is still uncertain [17, 18]. This could create anxiety in parents and unintentionally facilitate the family's search for information in inappropriate ways, such as the Internet, creating preconceptions that will lead to conclusions based on erroneous experiences and data. At the same time, however, communication of our diagnostic hypothesis ensures, first and foremost, transparency in the doctor-patient relationship, laying the foundation for a solid relationship of trust, which is fundamental in the treatment process. Otherwise, in fact, we would have to either pretend that we have no diagnostic hypothesis, which could create even more fear in parents seeing us as "disoriented", or admit that we do not want to share our hypothesis with them, creating a strong detachment between the medical team and the family. The communication of the uncertain diagnosis, instead, must be part of a truthful and empathetic dialogue between the physician and parents, where there can be time to cut back their fears and provide the appropriate information for this stage, discouraging parents from using other means of information. Secondly, communicating the diagnosis when it is still uncertain ensures, a longer time frame for family to understand, to accept and to elaborate the diagnosis and to redirect goals. This happens because, while waiting for definitive confirmation, the family can consider the possibility of the disease while they still hold out hope, thus having time to make their own considerations and allowing the shock of the diagnosis to settle in [18, 22].

As clinicians, we need to remember that time for dialogue is time for cure: we need to dedicate time to answer all parents' questions and provide them with accurate and current information, including an overview of DS and referrals to local and national support groups [6, 9].

### Where to communicate the diagnosis?

It is essential to ensure a quiet and private setting for the dialogue. The diagnosis must be communicated to both parents at the same time, if possible, and not in the presence of other people such as visitors or roommates [6]. It is recommended to always communicate the diagnosis in presence, unless there are serious exceptions, avoiding telephone calls, which could be cold and detached and would not allow the physician to perceive the family's true feelings. The family should therefore be contacted simply to say that we have received the result of the genetic tests and that we would like to meet the parents to discuss the matter together.

### Who should communicate the diagnosis?

Most of our opinions and beliefs reflect what we have learned in the course of our clinical practice, so it is important that those talking to parents are doctors who are up-to-date on the syndrome and have professional experience with children with DS [8, 10]. For the same reason, it is preferable for gynaecologists and geneticists, during both pre- and post-natal diagnoses, to be accompanied by paediatricians and/or neonatologists with such experience who can discuss the child's possible future prospects, the therapeutic possibilities and the opportunities offered by today's society. This gives parents a multidisciplinary view of the condition and allows professionals to answer any questions about the present and future, that may arise when the diagnosis is communicated, in an in-depth and up-to-date manner [16]. According to several studies, as shown in Skotko's 2009 article, parents prefer the communication of the diagnosis to take place in the presence of a paediatrician [22].

The emotional difficulty experienced by the doctor himself at the thought of communicating such a diagnosis may erroneously lead him to have an overly optimistic attitude (a defensive reaction, known as *denial*), thus risking making the parent, who is experiencing sadness and discouragement at the news, feel guilty [8]. On the other hand, the doctor may have an extremely pessimistic attitude, immediately proposing termination of the pregnancy as a solution, without giving the parents time to process what has happened and express their doubts or wishes. The best attitude, therefore, is not to let our emotions show too much, but to be ready to welcome and legitimise the parents' emotions, aware that for some this may be "bad news" while for others it is experienced with greater serenity from the very beginning [8].

The fear, discouragement and disorientation that we as doctors may feel when faced with a diagnosis of disease is absolutely understandable and human; let us take a few moments to face our feelings and prepare ourselves for communication with the family. Not respecting this time may lead us to act impulsively, making communication less effective, and by appearing confused or frightened, we may not be the strong and understanding reference point that the family needs.

“C*ompassion for the parents is a feeling which every doctor must have. Anyone who can tell parents that their child is gravely affected in this way without, at the same time, feeling broken-hearted at the thought of their terrible anguish on hearing the news is not worthy of our calling.”*

- "Le Professeur Lejeune", Jean-Marie Le Méné

### Prenatal diagnosis: how to communicate it and how to accompany parents in the decision-making process.

As in all health care contexts, it is essential to create a good relationship of trust between doctor and patient so that parents feel supported in their decision-making process and feel they have the elements to make an informed choice. To this purpose, it is important that the doctor giving the news has a positive attitude and extensive knowledge of the condition [22]

The diagnosis must be communicated neutrally, i.e. without accompanying it with phrases that may imply a personal judgement, such as "I'm sorry to tell you that...", "I have bad news for you", etc... [8, 13]. The doctor must adopt an empathetic attitude towards the parents with a communication style that is sensitive and caring, but at the same time confident and clear, using simple language. Expressions that may be offensive and/or obsolete (e.g. Mongolism, etc.) must be absolutely avoided, and person-centred language must be used, referring to the child as a "child with Down Syndrome" and not as a "Down child".

Once the simple diagnosis has been communicated, it is helpful to stop and ask the parents what they know about the syndrome: this is a good way of being non-directive, sounding out the parents' reaction and setting up the best possible discussion afterwards [18].

After listening to the parents, the doctor should try to calmly answer their questions and concerns and then give them a brief summary of the features of the syndrome, trying to present them with a balanced view of the positive and negative aspects [10].

With regard to the positive aspects, we can refer to our own experience, if any, and to numerous articles that have investigated the quality of life of individuals with DS and their families. Actually, it is common for children with DS to arouse a climate of affective intensity greater than normal, so that some Authors have come to speak of a "gentleness gene" [3] or a "happy personality" [27]. Most parents stated that they love their son or daughter with DS and are proud of them, adding that these children have had a positive impact on their lives [4, 24]. From the point of view of people with DS, the overwhelming majority of them indicated that they live happy and fulfilling lives [23]. Overall, it has been shown that positive themes tend to dominate within modern-day families having members with DS, although challenges were not insignificant for some [20], and a significant number of parents admitted that raising a child with DS was not without challenges [4].

In dealing with the negative aspects, the greater care and attention that these children require must undoubtedly be made explicit, as well as the possibility for most of them to achieve not complete autonomy in adult life, although current society is increasingly implementing aids in this sense. Regarding the possible comorbidities, long and sterile lists of them, which risk making parents feel overwhelmed and powerless, should be avoided. In our opinion, when talking to parents about the comorbidities we should focus primarily on the specific features of their child and then talk about the general features, avoid listing comorbidity that, at this stage, we can exclude with certainty in the foetus. Talking about present or possible comorbidities, we recommend prioritize those that require life-saving treatments in the first few months after birth (formerly duodenal atresia, tetralogy of Fallot, interventricular defects, etc.), which might be highlighted during pregnancy, explaining their characteristics, the treatments available, and their implications. Secondly, we suggest to mention neurodevelopmental delay and intellectual disability, which may preclude the achievement of full autonomy, and, finally, the other minor comorbidities. Regarding the latter, it is not necessary to list them specifically, it is more correct to talk about them when, eventually, they are diagnosed in the child or when it is important to implement specific behaviours for their prevention (e.g., screening for sleep apnea or cervical X-ray for atlantoaxial instability, etc.) or, finally, in the case of direct question in this regard by the parents.

The description of the possible comorbidities should be always accompanied by a description of the possibile therapeutic strategies, when available, and of the currently achievable outcomes. It should also be made clear that intellectual disability is always present, as mentioned above, and the degree of retardation is variable and cannot be established prenatally. At the same time, the therapies available today, such as physiotherapy and speech therapy, have led to important results from the point of view of neurodevelopment and autonomy in children with DS [19].

We should remember to leave time for tears and not try to fill silences, rather it can be helpful to give parents a few moments to talk alone if they wish [18]. Bearing in mind the emotional and psychological impact that a diagnosis often have in the couple of parents it is fundamental in the communication of the diagnosis. As suggested in literature [11], a prenatal diagnosis of genetic disorder or other foetal abnormality is most cases unexpected and shocking for parents, who often experience a whirlwind of emotions of fear, anger, and grief, and feelings of mourning for the “hoped-for child” that require to be considered and accepted by professionals. These feelings and emotions, moreover, may also occur and accompany subsequent pregnancies.

Research results suggested that the emotions experienced by parents of children with DS are similar regardless of the time of diagnosis, whether it is prenatal or neonatal [15].

Parents who, after the first part of the counselling, feel that they cannot raise a child with DS should be informed of the two possible alternatives, both of which must be mentioned: adoption and termination of pregnancy [5, 10, 18]. These two proposals should only be made after having listened to the parents' thoughts on the diagnosis, since we must remember that these are usually desired pregnancies and therefore the proposal to terminate the pregnancy must be made with caution and delicacy [7]. It sometimes happens that parents choose to carry out invasive diagnostic tests with the sole intention of preparing themselves to welcome a baby and not with thoughts of terminating the pregnancy. It is therefore important never to take anything for granted in front of a medical report, but first to listen to the parents in order to understand their inclination and their fears or doubts [14].

The discussion about the different possibilities should be conducted in a non-directive way, without addressing parents according to our feelings and without proposing a solution in a preferential way [7, 21]. If, at the end of the counselling, the family is undecided about the choice to be made, it is recommended to propose a new meeting after a few days and offer psychological support if they wish. If the parents have difficulty in making a decision because of their lack of knowledge about the condition, it is a good idea to offer them the opportunity to meet other parents who already have children with DS, in order to give them a more in-depth view, while underlining the differences that exist from child to child.

To parents who, after the first part of the interview, feel they want to further explore the possibility of continuing the pregnancy, we must provide further information about the syndrome and, if they so wish, about the life expectancy and possibilities that today's society offers these children and their families. As doctors we must emphasise our willingness to support them in every need, providing further information on the follow-up programme offered by the hospital or nearby reference centres.

During counselling it is important to provide parents with contact details of local and national support groups, parents' associations and to offer them the opportunity, if they wish, to meet families who are already raising a child with DS, so that they can hear first-hand experience [6, 18].

Communicating the diagnosis does not end with the first meeting, but requires continuity of care, providing references for access to information, resources and help, and planning a new meeting with the family if they so wish [18].

We have to remember that, when the diagnosis is prenatal, the parents are faced with the decision between continuing or interrupting pregnancy in a time characterized by emotional distress and crisis, and this choice may be for many parents particularly challenging. In an extensive article by Angus J. Clarke and Carina Wallgren-Pettersson on ethics in genetic counselling [7], the authors emphasise how important it is to help parents project into the future about the choice they are going to make, as we can occasionally make decisions objectively and rationally, and then realise that the decision conflicts with how we feel. A decision made through a process of logical reasoning may then be unacceptable or even repugnant to ourselves. Our aim is therefore to help our patients understand what their decision means from a biological point of view, but also to help them project themselves into the future so that they can reach a decision with which they will live well in the long term.

Beyond the first moment and the acute responses to the diagnosis, it is important to consider that it is the beginning of a complex process that require the acquisition and assimilation of information about a condition that in the majority of cases was unconsidered, and it is often associated in change in parents expectations and life beliefs.

According to the study conducted by Skotko in 2009, the most important questions to which parents are seeking answers are: "what is Down Syndrome?", "what are the causes?" and "what does it mean for a family to have a child with DS, in practical terms?" [10, 22]. The latter question can be confidently and competently answered by a doctor who has experience with the syndrome and the lives of affected patients and their families, but even more important than the doctor in answering this question are the families who already have a member with DS in their midst [10, 22]. It is therefore essential that the doctor contributes to creating a network among the families he or she assists, both those who already have children with DS and those who have just received their child's pre- or post-natal diagnosis and are interested in learning more. There are considerable benefits that families report from meeting and talking to each other, in terms of encouragement, awareness and informed choice. At the same time we need to prepare families who already have children with DS, and who make themselves available to meet new expectant families, for the fact that the families they meet may still choose to terminate the pregnancy after talking to them and their child. It may help to ask them how they might react if this were to happen and whether they feel ready to offer this dialogue service in any case.

In conclusion there is no doubt that the moment of the communication of the diagnosis should be only the first moment in accompanying and support parents in the process of sense-making about the diagnosis and decision-making. Usually, the prenatal period and the first months after birth are the most difficult from a psychological point of view for parents. On the one hand, in fact, the process of acceptance of the condition is underway and fears with respect to the disease and its implications are greater, but on the other hand, the bond with the child is still forming and can therefore be more fragile. In addition, these are often the most complex months from a medical point of view, because of the need for surgery, for example, or because of the increased frequency of infections, which are more complex to manage in the newborn. Therefore, in this period it will be essential, as physicians, to show our presence, give our utmost availability and remember not only to care about the health of the child but also to ask the parents, at every meeting opportunity, how they are doing and to continue, if we deem it appropriate, to offer them psychological support or meeting with associations and families, even if at first they had refused them. Let us always remember that in these early stages parents' emotions and thoughts can be extremely changeable, and it will be important to always welcome their fears and struggles, without making them feel "wrong," because they are simply "human."

Family’s adaptation to the child’s disability and special needs is a lifelong process, and professional support and care should be not limited to the first months or years of life; however, generally, as time goes on, fears spontaneously decrease while the bond of affection with the child grows and itself becomes a support for the parents in difficulties.

### Postnatal diagnosis: how to communicate it and how to provide the family with adequate support.

Regarding how to communicate the diagnosis of a malformative syndrome in the postnatal phase, the 2021 article by Serra and colleagues [17] already provides important suggestions, and thus we refer to it for a broad and in-depth analysis of the topic. In our article, given the specificity of the subject matter, in addition to summarizing the main general points, we include practical examples (phrases, tips, ...) that relate more specifically to DS, while also recalling some articles that can help physicians to provide adequate and up-to-date information with respect to the syndrome and the quality of life of our patients.

When communicating, first of all it is important to know that families generally do not want to feel pitied with unprofessional phrases such as "I'm sorry to tell you that your child has Down Syndrome", but instead like to be congratulated on the birth of the child [8, 9, 24]. During the first conversation for the communication of the postnatal diagnosis it is good for the child to be present and the doctor must refer to him/her by name and speak in person-centred language, emphasising that the child has Down syndrome and not that he/she is a "Down child" [18]. Every child with DS is unique and will have characteristics of the mother and father, just like all other children, to whom he/she will be much more similar than different [10, 18].

Afterwards, it is good to ask parents what they know about this syndrome and subsequently summarize its main aspects briefly and clearly. The information we provide should be balanced, illustrating the more challenging aspects of the syndrome (better not to call them "negative aspects" here) and the positive aspects. In the postnatal phase, our attitude may be more positive and encouraging than recommended in the prenatal one, where impartiality must prevail; this may help parents overcome the initial shock and increase their self-confidence. Be careful, however, not to give false hope or downplay the fears and dejection that parents may feel.

Regarding the challenging aspects, the main complications and comorbidities should be explained, but, as mentioned already, it is good to avoid long, detached lists and rather focus on the child in front of us, talking about his comorbidities without making general and misleading speeches (e.g. if the child does not have gastrointestinal malformations there is no reason to list them to the parents). The discussion of the comorbidities should be accompanied by a description of the therapeutic possibilities that exist today and of the achievable outcomes (e.g. cardiac and gastrointestinal malformations can all be corrected surgically). With regard to other comorbidities to which they may be predisposed, we can reassure the parents by informing them that the child will be included in follow-up programmes at specialist centres with the aim of identifying any problems at an early stage in order to provide appropriate measures as soon as possible. It is not necessary to give all the information at the first meetings: during the follow up the parents will be gradually informed about what they should pay attention to during the different phases of their child's life.

It should also be pointed out that children with DS reach most of the developmental milestones later than other children, but almost all of them are still able to speak, walk and achieve a good level of independence, being able to attend all social environments, from school to sport [24]. In this respect, physiotherapy and speech therapy can help considerably, enabling children with DS to reach their developmental milestones early, with positive effects on their cognitive development. In addition, although caution should be used to not generate false hopes in the parents, it could be pointed out to them that research on the biological mechanisms of intellectual disability in DS has revived in the last 10 years. Many researchers are going to propose clinical trials to verify positive effects of rationally-based pharmacological interventions on the psychomotor development and cognitive skills of children with DS [1, 2, 26]. This could give to the parents some perspectives in the long term.

Regarding the positive aspects, we can tell parents about how these children are able to create around them a climate of affective intensity greater than normal, thanks to their "happy personality" and give examples of what some parents report. E.g. in the 2013 article of Kaposy, a parent, with respect to his child with DS, says: "He has some cognitive and physical delays, but his friendliness, his happiness and his sociability overshadow these delays when you meet him. We cannot imagine our family without him" [12]. It is important further to inform them that the vast majority of people with Down syndrome reported living happy and fulfilling lives [23] and that currently their life expectancy is up to 60 years [5].

It is important in this setting to emphasise our helpfulness and what we, as clinicians, can offer in terms of care and support.

Here is an example of a conversation: 'I visited your child and he is fine. I noticed some characteristics [*show them in simple language*] that made me suspect that N (*child's name*) might have Down syndrome. [*pause to observe parents' reactions*] What do you know about this syndrome?". Then briefly explain the physical characteristics and clinical implications of the syndrome and provide parents with references for further knowledge of the disease and all that society offers in terms of associations and services [8].

During these first and subsequent visits, we recommend paying attention to the child's strengths and achievements, helping parents to focus on the child's potential rather than his or her weaknesses [17]. These small attentions, combined with calling the child by name [25], for example, can help parents see their child beyond the illness and promote a healthy and welcoming parent-child relationship. It is also essential to put these parents in touch, if they so wish, with other families with children with the syndrome and with the local associations and support groups, so that they can share information and experiences and expand the support network, both for the family and the child [24].

It can be of great help at this stage to ask parents how they plan to communicate the diagnosis to siblings, relatives and friends, and to discuss it together if necessary. In cases where this is appropriate, it is also advisable to offer psychological support provided by the hospital.

## Conclusions

As a conclusion to this article we would like to leave a summary table (Table 1) of the most important recommendations regarding the communication of the diagnosis of DS, considering it a useful tool for professionals who are about to conduct such a delicate conversation. Rereading these key points each time before facing the moment of communication helps the physician to perform this task in the most appropriate way and to face with greater confidence the fears and the uncertainties that commonly arise in such a circumstance even in the most experienced professional

**Table 1:** Resume of the indication for a communication of the diagnosis

**When**
- Communicate the diagnosis as soon as it is reached and inform parents even when the diagnosis is still uncertain - Communicate to both parents at the same time - Dedicate time to answer all parents' questions
**Where** - Communicate the diagnosis in presence - Quiet and private setting
**Who**
- Doctors who are up-to-date on the syndrome and have professional experience - It is preferable for gynaecologists and geneticists to be accompanied by paediatricians and/or neonatologists
**How** *Prenatal diagnosis* - Have a positive attitude and extensive knowledge of the condition - Communicates the diagnosis without implying personal judgements - Absolutely avoid expressions that may be offensive and/or obsolete - Use person-centred language - Once the diagnosis has been communicated, stop and ask parents what they know about the syndrome - Balance positive and negative aspects - Inform and briefly describe to parents all three possible choices (continue or terminate the pregnancy and adoption) - Conduct the discussion in a non-directive way - Propose a new meeting after a few days - Offer parents the opportunity to meet other parents - Provide parents with contact details of local and national support groups- *Postnatal diagnosis* - Congratulate the parents on the birth of their child - It is good to communicate the diagnosis to the parents with the child present; refer to him/her by name and speak in person-centred language - Avoid offensive language and vague or approximate information - The discussion of the comorbidities should be accompanied by the therapeutic possibilities and the achievable outcomes - Provide balanced information - Emphasise your helpfulness and what you, as clinicians, can offer in terms of care and support. Explains to parents the follow-up programmes that are available for their child. - Offer parents the opportunity to meet other parents - Provide parents with contact details of local and national support groups

## Data Availability

Not applicable.

## Abbreviation

DS: Down Syndrome
